# Identification of Nonsynonymous SNPs in Immune-Related Genes Associated with Pneumonia Severity in Pigs

**DOI:** 10.3390/genes15081103

**Published:** 2024-08-21

**Authors:** Hiroki Shinkai, Kasumi Suzuki, Tomohito Itoh, Gou Yoshioka, Takato Takenouchi, Haruki Kitazawa, Hirohide Uenishi

**Affiliations:** 1 National Institute of Animal Health, National Agriculture and Food Research Organization (NARO), Tsukuba 305-0856, Japan; sinkai@affrc.go.jp; 2 Swine and Poultry Research Department, Gifu Prefectural Livestock Research Institute, Seki 501-3924, Japan; suzuki-kasumi@pref.gifu.lg.jp (K.S.); yoshioka-go@pref.gifu.lg.jp (G.Y.); 3 Food and Feed Immunology Group, Laboratory of Animal Food Function, Graduate School of Agricultural Sciences, Tohoku University, Sendai 980-8572, Japan; haruki.kitazawa.c7@tohoku.ac.jp; 4 Livestock Immunology Unit, International Education and Research Center for Food Agricultural Immunology (CFAI), Graduate School of Agricultural Sciences, Tohoku University, Sendai 980-8572, Japan; 5 Maebashi Institute of Animal Science, Livestock Improvement Association of Japan, Maebashi 371-0121, Japan; titoh@liaj.or.jp; 6 Institute of Agrobiological Sciences, National Agriculture and Food Research Organization (NARO), Tsukuba 305-8634, Japan; ttakenou@affrc.go.jp

**Keywords:** disease resistance, mycoplasmal pneumonia of swine, pattern recognition receptors, porcine pleuropneumonia, single nucleotide polymorphism, swine

## Abstract

We previously showed that several polymorphisms in genes encoding pattern recognition receptors that cause amino acid substitutions alter pathogen recognition ability and disease susceptibility in pigs. In this study, we expanded our analysis to a wide range of immune-related genes and investigated polymorphism distribution and its influence on pneumonia in multiple commercial pig populations. Among the polymorphisms in 42 genes causing 634 amino acid substitutions extracted from the swine genome database, 80 in 24 genes were found to have a minor allele frequency of at least 10% in Japanese breeding stock pigs via targeted resequencing. Of these, 62 single nucleotide polymorphisms (SNPs) in 23 genes were successfully genotyped in 862 pigs belonging to four populations with data on pneumonia severity. Association analysis using a generalized linear mixed model revealed that 12 SNPs in nine genes were associated with pneumonia severity. In particular, SNPs in the cellular receptor for immunoglobulin G *FCGR2B* and the intracellular nucleic acid sensors *IFI16* and *LRRFIP1* were found to be associated with mycoplasmal pneumonia of swine or porcine pleuropneumonia in multiple populations and may therefore have wide applications in the improvement of disease resistance in pigs. Functional analyses at the cellular and animal levels are required to clarify the mechanisms underlying the effects of these SNPs on disease susceptibility.

## 1. Introduction

Infectious diseases are the most serious threat to pig farm operations. Conventional countermeasures, such as vaccines and hygiene control, have limitations, and the use of antibiotics is becoming increasingly restricted worldwide because of the risk of the emergence of drug-resistant bacteria. Therefore, the genetic improvement of disease resistance in pigs is an urgent issue. There are very few examples of genome-wide association studies (GWAS) for disease resistance traits. Molecular breeding using genome editing has been shown to be effective, for example, by producing *CD163* knockout pigs that show no clinical signs against infection with porcine reproductive and respiratory syndrome virus (PRRSV) [1]. However, this practice has not been widely adopted owing to issues with consumer acceptance.

Pattern recognition receptors (PRRs) recognize many types of pathogen-associated molecular patterns (PAMPs) and induce host immune responses during the early stages of infection [2,3]. We have previously demonstrated that single nucleotide polymorphisms (SNPs) causing amino acid substitutions are abundant in PRRs such as Toll-like receptors (TLRs) and nucleotide oligomerization domain (NOD)-like receptors in pig populations [4]. Some of these polymorphisms have been confirmed to alter PAMP recognition in cultured cells and influence symptoms following experimental infection, vaccine response, and farm survival [5]. For example, A SNP (1205C > T) in *TLR5* reduces the recognition of *Salmonella*-derived flagellin in cultured cells and exacerbates diarrhea after experimental *Salmonella typhimurium* infection [6]. An SNP (2197A > C) in *NOD2* increased the recognition of muramyl dipeptide in cultured cells, a component of peptidoglycan in Gram-negative and Gram-positive bacteria, and reduced mortality in pigs in farms infected with porcine circovirus type 2 [7]. Two SNPs (1922G > A and 2752G > A) in *NOD1* were found to reduce the recognition of diaminopimelic acid in cultured cells, another component of the peptidoglycan, and the latter (2752G > A) significantly increased tissue colonization of *Salmonella* in a farm in Canada [8]. Furthermore, these SNPs were associated with pneumonia severity in a farm with poor hygiene [5]. In particular, *TLR5*-1205T and *NOD2*-2197C, respectively, increased and decreased the slaughterhouse pleuritis evaluation system (SPES) score, which is an index of lesions caused by *Actinobacillus pleuropneumoniae* (App), and *NLRP3*-2906G decreased the Goodwin’s lung lesion (GW) score, which is an index of lesions caused by *Mycoplasma hyopneumoniae* (Mhp) [5]. These findings indicate the possibility of pig breeding for disease resistance. The allele frequency of the above SNPs in Japanese commercial pig populations is being investigated, and their use as DNA markers for pig selection is being explored.

This study aimed to expand the research target to include a wide range of immune-related genes and search for candidate functional SNPs associated with pneumonia severity. We focused on nonsynonymous SNPs stored in the pig genome database. The target genes include PRRs that are involved in the recognition of nucleic acids derived from pathogens [9,10], which we have not yet studied, and receptors for molecules involved in opsonization, such as antibodies and complements [11,12], scavenger receptors [13], tripartite motif (TRIM) proteins that play important roles in innate immune signaling pathways [14,15], and transmembrane (TMEM) proteins, many of which have unknown functions but may be related to cancer and infectious diseases [16]. SNPs causing amino acid substitutions within these genes were extracted from the latest National Center for Biotechnology Information (NCBI) genetic variation database (dbSNP) [17], and an association analysis was performed on 862 pigs from four commercial farms with records of pneumonia severity.

## 2. Materials and Methods

### 2.1. Pig Populations and Measurement of Trait Data

Three-way crossbred ([Landrace × Large White] × Duroc) pigs maintained on two pig farms (designated as farms A and B) from June 2016 to August 2017 and two pig farms (designated as farms A and C) from June 2019 to March 2020 in Gifu Prefecture, Japan were used in this study (Table 1). The pigs sampled from 2019 to 2020 were the same as those used in our previous study, and pigs in farms A and C had the same genetic background [5]. The pigs in farms A and B were reared in semi-windowless swine barns with an all-in/all-out production system. In contrast, pigs in farm C were reared in open barns, which did not adopt all-in/all-out production at the entire barn level. Feeding, vaccination, sampling, and trait measurements were performed as previously described [5]. The GW score is an index of the degree of hepatized lung lobes caused by Mhp on a scale of 0 to 55 points [18], and its logarithmically transformed value was used as a continuous variable in the subsequent association analysis [19,20]. The SPES score is an index that evaluates adhesion between the lung and diaphragm caused by App on a scale of 0 to 4 points [21,22,23], and was used as an ordinal scale in the subsequent association analysis. The GW score was measured for all farms, but the SPES score was not measured for farms from 2016 to 2017 and was only measured for farms from 2019 to 2020.

### 2.2. Targeted Resequencing

The information of polymorphisms causing amino acid substitutions in pig immune-related genes was extracted from the NCBI dbSNP database (Build 150) based on Sscrofa 10.2 genome sequence [24]. Polymerase chain reaction (PCR) primers used to amplify genomic fragments containing polymorphic sites were designed using DesignStudio software v8.0.0 (http://designstudio.illumina.com, accessed on 31 July 2024). Genomic DNA was extracted from Japanese breeding stock pigs, particularly, from the tails of 82 Duroc pigs from Gifu Prefecture, and 60 Duroc and 32 Landrace pigs from another prefecture, using a DNeasy Blood and Tissue Kit (QIAGEN, Hilden, Germany). Multiplex PCR and library preparation were performed using AmpliSeq Library PLUS for Illumina and AmpliSeq CD Indexes Set A for Illumina according to the manufacturer’s instructions, and sequencing was performed on an Illumina MiSeq platform (Illumina Inc., San Diego, CA, USA). Primer sequences and low-quality regions in the sequencing reads were trimmed using the Trimmomatic version 0.36 at default settings [25]. Processed sequencing reads were aligned to the pig genomic sequence (Sscrofa 10.2) using Burrows-Wheeler aligner and Picard 2.18.7 (https://broadinstitute.github.io/picard/, accessed on 31 July 2024). Polymorphisms such as SNPs, insertions, and deletions were detected using GATK 3.8.1 [26,27].

### 2.3. Genotyping

DNA was extracted from lung tissues of 862 three-way crossbred pigs with trait data. SNP genotyping was performed using the Fluidigm SNP Type Assay and the 96.96 Dynamic Array IFC (integrated fluidic circuit) controller HX, FC1 thermal cycler, and EP1 System (Standard BioTools Inc., South San Francisco, CA, USA) according to the manufacturers’ instructions. The assay was based on an allele-specific PCR detection system, including allele-specific forward primers labeled with the fluorescent dye FAM or VIC and gene-specific reverse primers. Genotypes for each SNP were determined by analyzing the data using Fluidigm SNP Genotyping Analysis software v4.5.1.

### 2.4. Data Analysis

All the statistical analyses were performed using SPSS version 26 (IBM Corp., Armonk, NY, USA). Correlations between traits were evaluated using Pearson product-moment correlation coefficients. Univariate analysis of the association between each polymorphism and trait for each group divided by year and farm was performed using analysis of variance for continuous variables and the Kruskal–Wallis H test for ordinal scales. Polymorphisms that may be associated with traits in the univariate analysis (*p* < 0.2) were extracted, and multivariate analysis of the association between polymorphisms and traits was subsequently performed using a generalized linear mixed model (GLMM), with sex and sampling date as random effects (*p* < 0.05). The logarithmically transformed GW score and the SPES score were assumed to follow Gaussian and multinomial distribution, respectively. To avoid multicollinearity in the GLMM analysis, haplotype inference was performed using SNPAlyze V9.0 (Dynacom, Chiba, Japan).

## 3. Results

### 3.1. Characteristics of Pig Populations and Traits

**Figure 1:**
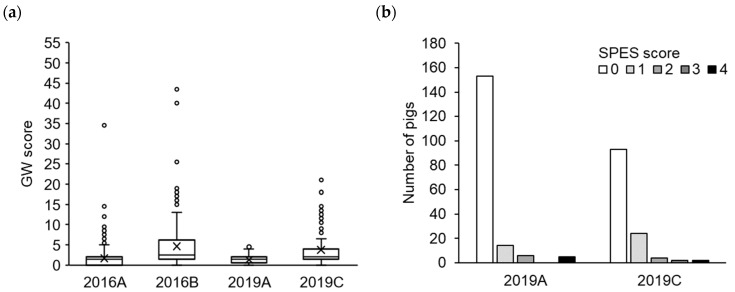
Comparison of lung lesion scores between pig populations. Sample variability is shown as box plots for GW score (**a**) and by frequency distribution of each SPES score (**b**). In the box plots, boxes represent the 25th and 75th percentiles, horizontal lines within the box represent the median values, and crosses represent the mean values. The whiskers represent the lowest and highest values in the 25th percentile minus 1.5 times the interquartile range (IQR) and 75th percentile plus 1.5 times the IQR, respectively, and the values outside this range are shown as circles.

Figure 1 shows the pneumonia severity scores of the four populations according to year and farm. The GW scores were lower in farm A and higher in farms B and C, where hygiene conditions were considered poorer (Figure 1a). No significant difference was found in the GW scores between years. The SPES score data were not available for 2016, but the symptoms were more severe in farm C than those in farm A in 2019, consistent with the GW scores (Figure 1b). No significant correlation was observed between the GW and SPES scores in farms 2019A and 2019C (Table S1).

### 3.2. Extraction and Narrowing Down of Target Genes and Polymorphisms

Our previous studies have revealed that several amino acid polymorphisms in PRRs affect pathogen recognition and clinical symptoms in pigs [5]. These results suggest that functional SNPs with amino acid substitutions associated with disease resistance are likely to be found in other PRRs and immune-related genes. We extracted candidate functional SNPs from over 60,000 nonsynonymous variants registered in the pig genome database [17] and selected 634 polymorphisms in 42 genes, including 14 PRRs, 6 receptors for molecules involved in opsonization, 2 scavenger receptors, 2 lectins, 5 TRIM proteins, 8 TMEM proteins, and 5 other immune genes (Table S2).

To investigate the diversity of these polymorphisms in Japanese breeding stock pigs, we performed targeted resequencing of two breeding stocks of Duroc pigs and one breeding stock of Landrace pigs. The results revealed that 80 polymorphisms in 24 genes had a minor allele frequency of 10% or more in any breeding stock (Table S3). To conduct an association analysis between the polymorphisms narrowed down using pure breeds and pneumonia severity, these polymorphisms were genotyped in three-way crossbred pigs. A total of 62 polymorphisms in 23 genes were successfully genotyped and used for subsequent analyses (Table S4). There were two polymorphisms for which PCR primers could not be designed, 12 for which genotyping was difficult owing to poor cluster separation after PCR, and four for which the genotype was fixed (Table S3).

### 3.3. Association between Polymorphisms in Immune-Related Genes and Traits

First, we evaluated SNPs, namely the 62 polymorphisms mentioned above and five SNPs in our previous study (*NLRP3*-2906, *NOD2*-2197, *TLR5*-1205, and *NOD1*-1922/2752) [5], which are potentially associated with traits in each population according to year and farm using univariate analysis (*p* < 0.2; Table S5). If multiple SNPs that could be associated with a trait were detected within a single gene, the haplotypes consisting of these SNPs were inferred (Tables S6 and S7). Subsequently, the association of these SNPs and haplotypes with traits was analyzed using multivariate analysis based on the GLMM, considering the effects of sex and sampling date (*p* < 0.05; Tables S8 and S9).

**Figure 2:**
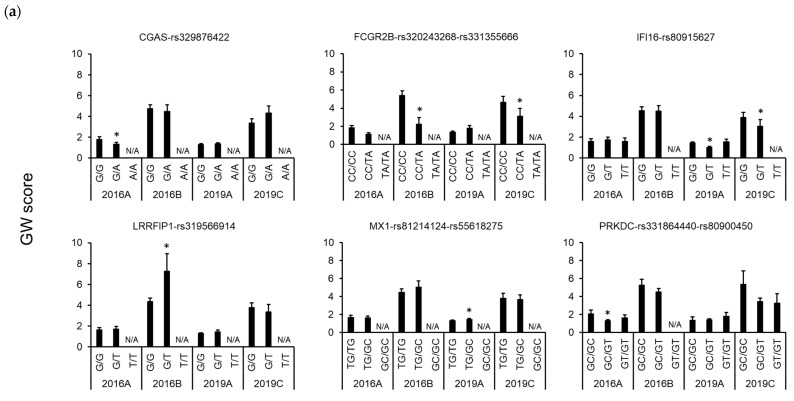

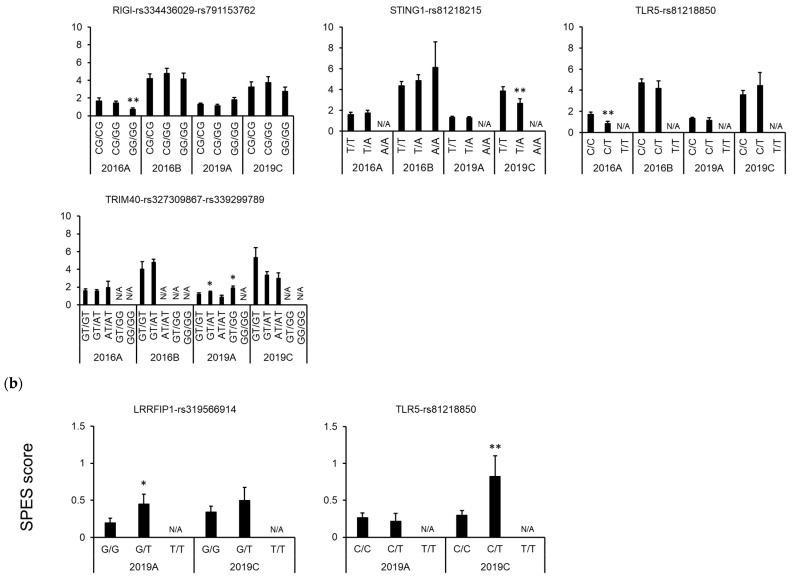
Comparison of the effects of genes and polymorphisms affecting the GW (**a**) and SPES (**b**) scores among pig populations. Means and standard errors of phenotypic value are shown. Significant differences from homozygous individuals for the most frequent allele on the left end detected by association analysis using generalized linear mixed model are indicated using asterisks (* *p* < 0.05, ** *p* < 0.01). Genotypes for fewer than five animals are indicated as N/A.

Five SNPs, namely *FCGR2B*-rs320243268C/T-rs331355666C/A, *IFI16*-rs80915627G/T, *LRRFIP1*-rs319566914G/T, and *STING1*-rs81218215T/A, that were significantly associated with the GW score were detected in farms B and C, which had poor hygiene conditions compared with farm A (Figure 2a and Table S8). Although the GW score in farm A was low, which was not a problem in the farm, it was associated with eight SNPs: *CGAS*-rs329876422G/A, *MX1*-rs81214124T/G-rs55618275G/C, *PRKDC*-rs80900450C/T, *RIGI*-rs334436029C/G, *TLR5*-rs81218850C/T, and *TRIM40*-rs327309867G/A-rs339299789T/G.

In terms of the SPES score, two SNPs, *LRRFIP1*-rs319566914G/T and *TLR5*-rs81218850C/T, were significantly associated with lesion severity in farms A and C in 2019, respectively (Figure 2b and Table S9). For *TLR5*-rs81218850C/T, opposite effects on GW and SPES scores were detected in the two farms with different hygiene conditions (Figure 2); therefore, further analysis using data from other farms is required to clarify the true effect of this SNP.

Table 2 summarizes the alleles and amino acids considered resistant or susceptible to mycoplasmal pneumonia of swine (MPS) and porcine pleuropneumonia. *FCGR2B*-rs320243268T-rs331355666A was considered a resistance allele because it lowered the GW score in the two farms with poor hygiene conditions (Figure 2a). In addition, *IFI16*-rs80915627T was considered a resistance allele because it lowered the GW score of the two farms in 2019. Meanwhile, *LRRFIP1*-rs319566914T was considered a susceptibility allele because it increased both GW and SPES scores in two farms at different years with different hygiene conditions (Figure 2). Association analysis of a farm with severe symptoms indicated that the *STING1*-rs81218215A allele was likely resistant to MPS (Figure 2a). The allele frequencies of the five SNPs were similar among the four populations, with no major differences (Table S4). For *LRRFIP1*-rs319566914G/T, the frequency of the resistance allele was high, whereas for *FCGR2B*-rs320243268C/T-rs331355666C/A, *IFI16*-rs80915627G/T, and *STING1*-rs81218215T/A, the frequency of the susceptibility alleles was high in the study population (Table 2). The *FCGR2B*-rs320243268C/T also had high values of *H_e_* and *N_e_*, which are indexes of genetic diversity. The other seven SNPs, namely *CGAS*-rs329876422G/A, *MX1*-rs81214124T/G-rs55618275G/C, *PRKDC*-rs80900450C/T, *RIGI*-rs334436029C/G, and *TRIM40*-rs327309867G/A-rs339299789T/G, were suggested to be associated with the GW score in farm A, where the symptoms were relatively mild. Further analysis of other farms is required to properly determine the effect of each allele.

## 4. Discussion

We have previously demonstrated that the amino acid polymorphisms *TLR5*-1205C/T, *NLRP3*-2906A/G, and *NOD2*-2197A/C in PRRs in pigs, which have been shown to alter pathogen recognition and cytokine production at the cellular level, are also associated with pneumonia severity in commercial pig farms [5]. In this study, we expanded our focus to a wide range of immune-related genes, including PRRs, and analyzed their association with pneumonia severity by adding data from two different farms (2016A and 2016B) to previously studied farms (2019A and 2019C). In the association analysis, all genotyped SNPs were entered into multivariate analysis based on GLM in a previous study [5]; however, in this study, SNPs that were likely to be associated with the traits were first narrowed down using univariate analysis, followed by multivariate analysis. *NLRP3*-2906A/G, which was significantly associated with the GW score in 2019C farm in a previous study [5], did not show any significant difference in this study. This may be because polymorphisms showing stronger associations with the GW score, such as *FCGR2B*-rs320243268C/T-rs331355666C/A, *IFI16*-rs80915627G/T, and *STING1*-rs81218215T/A, which were not used in the previous study, were incorporated into the analysis model in the present study. In contrast, for *NOD2*-2197A/C and *TLR5*-1205C/T, which were previously found to be significantly associated with the SPES score in the 2019C farm [5], the significant association between *NOD2*-2197A/C and the SPES score disappeared and that between *TLR5*-1205C/T and the SPES score was detected by incorporating polymorphisms in other immune-related genes into the analytical model in this study.

We detected nine additional genes associated with pneumonia severity. Among these, *FCGR2B* was associated with the GW score in two severely affected farms and could be a reliable genetic marker for improving resistance to MPS in pigs. Fc-gamma receptor II B (FcγRIIB) is the only FcγR that sends inhibitory signals to cells through the immunoreceptor tyrosine-based inhibitory motif in its intracellular signaling domain, and the rare in-frame deletion Asn106del has been reported to abolish immunoglobulin G (IgG) binding to FcγRIIB in humans [28]. In pigs, a specific FcγRIIB activation in alveolar macrophages significantly increased the production of interferon (IFN)-α and IFN-β in response to PRRSV infection, but no polymorphisms affecting function are known thus far [29]. rs320243268 and rs331355666, which were found to be associated with GW score in this study, are located in the extracellular domain of FcγRIIB, similar to human Asn106del, and may therefore affect IgG binding.

The GW score was significantly associated with interferon gamma-inducible protein 16 (*IFI16*) in two farms and *STING1* in a severely affected farm. STING1 (stimulator of interferon response cyclic guanosine monophosphate-adenosine monophosphate (cGAMP) interactor 1) plays an essential role in the innate immune response to DNA derived from viruses and bacteria, and is a key adaptor for the intracellular DNA-sensing pathway [30]. STING is recruited to IFI16 following recognition of double-stranded (ds) DNA by IFI16 to induce type I IFN production [31]. cGAS (cGAMP synthase), another DNA sensor, synthesizes cGAMP from ATP and GTP upon encountering DNA in the cytoplasm, which then binds to STING to induce IRF3-mediated IFN-β production [32,33]. The cGAS-STING signaling pathway is also being actively studied in pigs and is activated by PRRSV and porcine circovirus type 2 infections [34,35]. IFI16 has also been reported to be related to PRRSV and pseudorabies viral infections, but the reports are few compared with those of the cGAS-STING signaling pathway in pigs [36,37]. Interestingly, pig IFI16 has an extra pyrin domain that is absent in humans [38]. The amino acid polymorphism caused by *IFI16*-rs80915627, which was found to be associated with the GW score in this study, is located in this extra pyrin domain and may also be related to differences in the species-specific roles of IFI16.

*LRRFIP1* (leucine-rich repeat flightless-interacting protein 1) was associated with the GW score in a severely affected farm and the SPES score in a mildly affected farm, and it could be a reliable genetic marker for improving pneumonia resistance in pigs. LRRFIP1 is a cytosolic nucleic acid-binding protein that recognizes both dsRNA and dsDNA and is known to produce IFN-β upon infection of the host with vesicular stomatitis virus and *Listeria monocytogenes* [39]. Although GWAS analyses have reported that *LRRFIP1* is associated with adiposity, inflammation, schizophrenia, and bipolar disorder in humans and lameness disorders in cattle, no studies have been reported in pigs [40,41,42].

The genetic polymorphisms identified in this study can be applied in the improvement of disease resistance in pigs. For this, the effects of these polymorphisms on gene function should be biologically confirmed at the cellular and animal levels, and allele frequency in the population must also be considered. The frequency of the resistance allele of *LRRFIP1*-rs319566914 was nearly 90%, and it may be possible to fix this allele in commercial Japanese pig populations through artificial selection. In contrast, for *FCGR2B*-rs320243268-rs331355666, *IFI16*-rs80915627, and *STING1*-rs81218215, susceptibility alleles were more frequent than resistance alleles in the population; therefore, active artificial selection may improve disease resistance. Because the genomic distance between *FCGR2B* and *IFI16* is approximately 2.5 Mb on chromosome 4, an analysis considering the linkage and haplotypes between these genes may be necessary.

Although further analysis in other farms is required owing to the results from farms with mild symptoms that are not actually a problem, significant associations of the GW score with *CGAS*, *MX1*, *PRKDC*, *RIGI*, *TRIM40* were detected. The effect of the polymorphisms in *CGAS* on disease resistance, including their interaction with *IFI16* and *STING1*, as mentioned above, should be analyzed. In pigs, the 11 bp deletion allele of *MX1*, located in the 1964–1974 in coding sequence and specific to the Landrace breed, increases viral proliferation after influenza A virus infection in NIH3T3 cells [43]. However, the two amino acid polymorphisms associated with susceptibility to the GW score in this study are located at positions 867 and 923 in the coding sequence and are far away from the 11 bp deletion site. PRKDC (protein kinase, DNA-activated, catalytic polypeptide), also known as DNA-PK, plays an essential role in nonhomologous end joining, which repairs DNA damage within the nucleus of host cells [44]. PRKDC also functions as a PRR that recognizes DNA and viruses in the cytoplasm and induces type I IFN production through STING [45]. RIG-I, a retinoic acid-inducible gene-I, is involved in the recognition of viral RNA. TRIM40 promotes the proteasomal degradation of RIG-I through ubiquitination to attenuate the innate antiviral immune response [9,46]. Therefore, the effects of polymorphisms in these genes on disease resistance as well as their interactions should be analyzed.

## 5. Conclusions

We searched for polymorphisms causing amino acid substitutions that are significantly associated with pneumonia severity in four different pig populations, targeting a wide range of immune-related genes, including PRRs. We found that four polymorphisms in *FCGR2B*, *IFI16,* and *STING1* were associated with the GW score, an index of MPS, and that a polymorphism in *LRRFIP1* was associated with both the GW and SPES scores, the latter an index of porcine pleuropneumonia. In future research, we will increase the reliability of these polymorphisms as DNA markers to improve disease resistance by biologically clarifying their effects on gene function at the cellular and animal levels.

## 6. Patents

The SNPs associated with pneumonia severity identified in this study have been applied for a patent in Japan (P2024-052189).

## Figures and Tables

**Table 1:** Number of pigs used in this study by year, farm, sex, and sampling season.

Year	Farm	Sex	Sampling Season				Total
			Summer	Fall	Winter	Spring	
2016	A	Female	48	27	15	24	114
		Male	61	30	48	26	165
		Unknown	0	0	1	0	1
	B	Female	57	25	25	21	128
		Male	56	30	36	29	151
		Unknown	0	0	0	0	0
2019	A	Female	0	0	66	12	78
		Male	0	0	76	23	99
		Unknown	0	0	1	0	1
	C	Female	45	23	0	0	68
		Male	41	16	0	0	57
		Unknown	0	0	0	0	0

Spring, March to May; Summer, June to August; Fall, September to November; Winter, December to February.

**Table 2:** Resistance and susceptibility alleles of immune-related genes found to be associated with pneumonia severity.

Gene	Chromosome: Position	dbSNPID	Resistance				Susceptibility			*H_e_*	*N_e_*	Function
			Allele	Amino Acid	Frequency		Allele	Amino Acid	Frequency			
*CGAS*	1:92483865	rs329876422	A	Met	0.156		G	Val	0.844	0.263	1.357	PRR for cytosolic DNA
*FCGR2B*	4:88887587	rs320243268	T	His	0.260		C	Arg	0.740	0.385	1.625	Receptor for antibody heavy chain
	4:88892821	rs331355666	A	Ser	0.054		C	Ala	0.946	0.102	1.114	
*IFI16*	4:91393489	rs80915627	T	Asp	0.180		G	Ala	0.820	0.295	1.419	PRR for cytosolic DNA
*LRRFIP1*	15:137424903	rs319566914	G	Ala	0.885		T	Ser	0.115	0.204	1.256	PRR for cytosolic DNA
*MX1*	13:204856712	rs81214124	T	Glu	0.816		G	Ala	0.184	0.300	1.429	Involvement in antiviral activity
	13:204856656	rs55618275	G	Asp	0.816		C	Glu	0.184	0.300	1.429	
*PRKDC*	4:79757139	rs80900450	T	Met	0.420		C	Thr	0.580	0.487	1.950	PRR for cytosolic DNA; involvement in V(D)J recombination in T and B cells
*RIGI*	10:33900015	rs334436029	G	Cys	0.335		C	Ser	0.665	0.446	1.804	PRR for viral RNA
*STING1*	2:141360482	rs81218215	A	Asp	0.170		T	Val	0.830	0.282	1.393	PRR for cytosolic DNA
*TRIM40*	7:22713980	rs327309867	G	Ser	0.548		A	Leu	0.452	0.495	1.982	Involvement in intracellular protein degradation
	7:22713916	rs339299789	T	Ser	0.994		G	Arg	0.006	0.012	1.012	

Frequency shows the combined value for four populations (N = 862). *H_e_*, Heterozygosity. *N_e_*, Effective number of alleles. PRR, pattern recognition receptor.

## Data Availability

The data presented in this study are available upon request from the corresponding author.

## Supplementary Materials

The following supporting information can be downloaded at: https://www.mdpi.com/article/10.3390/genes15081103/s1, Table S1: Pearson correlation coefficient between traits for each population; Table S2: List of genetic polymorphisms in 42 immune-related genes causing 634 amino acid substitutions extracted as candidate functional SNPs; Table S3: List of genetic polymorphisms with a minor allele frequency of at least 10% in Japanese breeding stock pigs confirmed by targeted resequencing and their genotyping status by the Fluidigm SNP Type Assay; Table S4: Genotype frequency of polymorphisms used for association analysis in each population; Table S5: Univariate analysis of the association between single nucleotide polymorphisms in immune-related genes and lung lesion scores; Table S6: Allele frequencies of SNPs and haplotypes that are potentially associated with the Goodwin’s lung lesion score; Table S7: Allele frequencies of SNPs and haplotypes that are potentially associated with the slaughterhouse pleuritis evaluation system score; Table S8: Multivariate analysis of the association between polymorphisms in immune-related genes and Goodwin’s lung lesion score based on the generalized linear mixed model; Table S9: Multivariate analysis of the association between polymorphisms in immune-related genes and slaughterhouse pleuritis evaluation system score based on the generalized linear mixed model.
